# Diabetic Retinopathy and BDNF: A Review on Its Molecular Basis and Clinical Applications

**DOI:** 10.1155/2020/1602739

**Published:** 2020-05-18

**Authors:** Mehrdad Afarid, Ehsan Namvar, Fatemeh Sanie-Jahromi

**Affiliations:** Poostchi Ophthalmology Research Center, Shiraz University of Medical Sciences, Shiraz, Iran

## Abstract

Impairment of neuroprotection and vasculopathy are the main reasons for the progression of diabetic retinopathy. In this review, we decided to illustrate the molecular and clinical aspects of diabetic retinal neuro-vasculopathy. We searched the Web of Science, PubMed, and Scopus databases with these keywords: “brain-derived neurotrophic factor” and “vascular endothelial growth factor” and/or “diabetic retinopathy.” The most relevant in vitro and clinical trial studies were then extracted for final interpretation. Brain-derived neurotrophic factor and the vascular endothelial growth factor have pivotal roles in the pathogenesis of diabetic retinopathy. They have neuroprotective effects on the retina. However, there are controversial results on the relation between these two factors. Reviewing available articles, we have concluded that various concentrations of these molecules at different stages of retinopathy may exert different effects. Optimal doses of the brain-derived neurotrophic factor at the early stages of retinopathy may have a neuroprotective effect. In contrast, higher concentrations of brain-derived neurotrophic factor might induce inflammatory responses. Damage to the retinal cells due to metabolic alterations associated with diabetes and its consequence vasculopathy may also lead to changes in the ocular microenvironment and cytokines. Changes in cytokines result in the modification of neural cell receptors and the overproduction of vascular endothelial growth factor. It seems that controlling the optimal levels of neuroprotective molecules in the retinal tissue is the main step to halter diabetic retinopathy.

## 1. Introduction

Brain-derived neurotrophic factor (BDNF) is a neuroprotective factor that may have various effects on the pathogenesis of some neurologic disorders [1]. The effect of BDNF has also been studied in some retinal diseases. According to the known physiologic mechanisms of BDNF, the impact of this protein has been studied in age-related macular degeneration (AMD) [2, 3]. The level of serum BDNF is shown to be altered in patients with AMD compared to normal people [3]. Age-related macular degeneration is one of the main causes of blindness in the elderly population in the world [4]. In contrast diabetic retinopathy (DR) is considered to be an important cause of blindness in the active population (between 25 and 65 years old) [5]. Today there is a great interest to discover the pathogenesis and molecular mechanism of diabetic retinopathy and AMD. The focus of this study is to review documents related to DR. It seems that vasculopathy and impairment of neuroprotection underlie the progression of DR. Vascular endothelial growth factor (VEGF) is a well-known mediator of DR progression [6]. Several treatment strategies target VEGF to control the progression of retinopathy [7]. But the relationship between BDNF and VEGF and their regulatory effect on each other is needed to be investigated. The aim of this study is to review the documented reports on the role of BDNF and VEGF in pathogenesis and control of diabetic retinopathy and suggesting a relationship between these two factors.

## 2. Methods

In this study we searched Web of Science, PubMed, and Scopus English databases from 1990 to 2020 and extracted the most related English articles with these keywords: BDNF and VEGF, BDNF and diabetic retinopathy, VEGF and diabetic retinopathy, neuroprotection and diabetic retinopathy. While surveying the reports, experimental, molecular, and case-control studies, as well as clinical trials and reviews about this subject, were selected and appraised. Subsequently a hypothesis on the role of BDNF in the progression and management of diabetic retinopathy was established.

## 3. Results and Discussion

The most relevant published documents (both original and review articles) were used to extract helpful information about BDNF, VEGF, gene structure and polymorphism, their molecular pathways, and involvement in diabetic retinopathy. Recent studies about the available treatment for DR considering BDNF and VEGF were also surveyed in the present study. A summary of reports focusing on the role of BDNF and VEGF in retinal neuro-vasculopathy and their clinical and experimental application for treatment is presented in Table 1.

### 3.1. Molecular Basis of DR

Diabetes is a metabolic disease and is associated with alteration of metabolites in serum and disruption of homeostasis in the retina. This change could induce oxidative stress and results in a drop of neuroprotective factors [29] which leads to DR. Pathologically speaking, the main hallmark of DR is the degeneration of neural retina as well as the alteration of retinal blood vessels [8–10]. Retinal ganglion cells and amacrine cells are the first target cells affected by DR-induced apoptosis. The clinical sign of this apoptosis is the structural changes of the inner retinal layer and nerve fiber layer such as a reduced thickness of this layer diagnosed by optical coherence tomography (OCT) [30]. Data from in vitro studies, experimental animal models, and tests from patients suggest that damage to neurons begins even before the onset of vascular disease [29]. Regarding the clinical features of DR and alteration in neural retina and retinal vasculature, two important factors are under consideration in different studies including brain-derived neurotrophic factor (BDNF) and vascular endothelial growth factor (VEGF).

### 3.2. BDNF

BDNF is a small basic protein (with an isoelectric point of 9.6) [31] and a member of the neurotrophin family. It has a pivotal role in the development of neurons and their function. It is expressed at its highest level in the brain of humans. Besides its importance in neuronal development, different reports are available indicating the effect of BDNF on non-neuronal cells. BDNF is expressed at lower amounts in other organs such as liver, heart, and lung [11, 32]. BDNF is produced by neurons and glial cells in the retina [29]. RPE cells are also well-defined sources of BDNF production in the retina [12, 33]. The human BDNF gene locus is on chromosome 11p13. A common single nucleotide polymorphism (SNP) called Val66Met or rs6265 is identified in the BDNF gene. Cells with this missense change in the coding exon of BDNF have a substitution of methionine to valine at position 66 and show disturbance of some BDNF related cellular activities such as its secretion, trafficking, and processing. Additionally it has been reported that this SNP is associated with structural and functional diversities in the brain. Individuals with this SNP demonstrate a lower level of BDNF in serum. On the other hand, diabetic patients demonstrate lower BDNF content in their serum compared with nondiabetic people. It seems that there is an association between BDNF SNP and diabetes, but recently it has been reported that serum BDNF is not related to genetic polymorphisms within BDNF gene in diabetic patients [34]. Different reports are available on the association between various features of diabetes (such as cognitive impairment and depression) with BDNF Val66Met SNP [35, 36]. However there are controversial results on the association of BDNF SNP and diabetes itself. In a study by Krabbe et al., it was reported that there is no association between BDNF SNP and type 2 diabetes [37]. In another study of Korean adults by Park and Daily BDNF rs6265 polymorphism is reported to be associated with a lower risk of type 2 diabetes [38]. Yet no document is available on the relation of BDNF SNP and development of DR. Furthermore, it has been demonstrated that epigenetic modification of BDNF gene might have a significant impact on the progression of DR [39].

BDNF expression is precisely regulated at the level of transcription, translation, and post-translation. This active regulation of BDNF reflects the importance of its cellular content in its different functions [32]. At transcription level, BDNF regulation is performed through at least 4 promoters in rats and produces a heterogeneous content of BDNF transcript in terms of coding and noncoding exons [40]. The primary form of BDNF, proBDNF, undergoes cleavage by several proteases and produces three fundamental products [41]. The mature form of BDNF is a 14 kDa protein. BDNF and its precursor form, proBDNF, have adverse effects on neuron survival. While BDNF supports cell viability, proBDNF leads to neuron apoptosis. BDNF launches its neurotrophic signaling pathway through binding to TrkB tyrosine kinase receptor and leads to nerve cell survival, repair, and development. ProBDNF induces apoptosis through interaction with p75 and triggering the apoptosis signaling pathway [42, 43].

According to previous studies, BDNF seems to be a good diagnostic marker for the detection of diabetic retinopathy at early stages [13, 32]. Recently, it has been reported that BDNF level in serum and aqueous humor of DR patients decreases remarkably even before the appearance of clinical signs [13].

Several studies have shown that BDNF content of the cell is decreased at both protein and mRNA levels in streptozotocin-induced diabetic rat retina [14, 15, 42]. Patients' evaluation also showed that BDNF level in serum was remarkably reduced in nonproliferative and proliferative DR cases. It has been suggested that a reduced BDNF level in serum is an indicator of the development of DR [15]. A decrease in BDNF level can promote neurodegeneration in animal models [14, 16]. It seems that early retinal neuropathy has a positive correlation with reduced BDNF levels [44].

### 3.3. VEGF

VEGF or VEGF-A is a growth factor and belongs to the PDGF/VEGF growth factor. It is a homodimeric glycoprotein with a mass of 45 kDa [17]. VEGF gene is mapped on chromosome 6p21.1 with 8 exons and goes under different splicing to produce 4 isoforms in humans. VEGF-A with 165 amino acids is the main isoform responsible for angiogenesis involved development processes and pathology [45, 46]. It has been shown that a polymorphism in the 5′ UTR of VEGF gene (C (-634) G) can increase the risk for diabetic retinopathy [17]. RPE cells, Muller cells, and glial cells as well as pericytes and endothelial cells are known to be the chief source of VEGF secretion in the retina [46].

VEGF is supposed to have an important role in the initiation of DR especially at early stages [17]. It has been confirmed that VEGF expression can be stimulated under hypoxia conditions and ischemia [18, 19]. Hypoxia-inducible factor 1 (HIF-1) is a DNA binding protein produced in hypoxia conditions. HIF-1 can bind to the VEGF gene and initiates the upregulation of VEGF expression [47]. Then VEGF binds to tyrosine kinase receptors (VEGFR1 and R2) on endothelial cells and establishes PIP2, PI3K, and Ras pathways. The outcome of these signaling processes is increased production of nitric oxide and cell proliferation which ultimately leads to angiogenesis [46]. Although VEGF receptors are mainly expressed on endothelial cells, their presence is confirmed on some other types of cells such as retinal cells [48].

VEGF is another important molecule under the shade of DR. VEGF is recognized as a growth factor with neurotrophic effect and can induce ocular neovascularization [20]. VEGF is reported to have an elevated level of expression in the intravitreous and fibrovascular membrane of patients with proliferative DR [21, 49].

### 3.4. BDNF VEGF Interaction

Because BDNF and VEGF each have angiogenic and neurotrophic effects, finding their collaboration with each other is additionally important to scientists. Ongoing examinations have indicated that BDNF can stimulate VEGF-mediated angiogenesis. Different intermediate molecules have been found downstream of BDNF for regulating VEGF secretion. For example, in human umbilical vein endothelial cells, BDNF acts through oxidative stress and Akt activation angiogenesis [50] whereas, in chondrosarcoma cells, BDNF exerts its effects through TrkB/phospholipase C (PLC) and ERK 1/2 [11]. It has been demonstrated that BDNF-induced angiogenesis promotes fracture healing in osteoblasts [11]. In contrast to this report, an inverse relationship between BDNF and VEGF serum content in DR patients has been reported [15]. To interpret the discrepancy between the available reports, several points need to be considered; neurogenesis and angiogenesis are of certain pathways that should be involved to regenerate damaged cells. BDNF and VEGF have critical roles for promoting neuro- and angiogenesis; however, each of them is under the regulatory control of different signaling pathways. Ischemic conditions and decreased level of BDNF in DR leads to neurodegeneration, and release of proinflammatory cytokines [15, 51]. As a result, VEGF expression and angiogenesis are attenuated [52]. Angiogenesis provides the background for the recruitment of circulating neurotrophins to the site of injury and enhances the regeneration of damaged neurons. So increased VEGF in DR patients occurs as a response to inflammatory cytokines and facilitates neuroretinal regeneration. It has been demonstrated that supplementation of exogenous BDNF at the early stages of DR and treatment of diabetic animal models could downregulate VEGF, preserve blood-retinal barrier (BRB), and protect other neurons from degeneration [29].

It can be concluded that the VEGF BDNF correlation should be considered in association with the DR phase. The positive correlation of BDNF and VEGF has been confirmed, though in some pathogenic conditions this relation might be reversed which promotes neurotrophin supplementation and regeneration of injured cells.

### 3.5. BDNF-Based Treatment for DR

Diabetic retinopathy paves the ground for neurodegeneration and retinal neural apoptosis. Neural and glial tissues as well as retinal microvasculature are sensitive to hyperglycemia and annihilate in this condition. BDNF and VEGF have recently achieved great interest because of their role in neurogenesis. The retina in diabetic patients suffers from neural cell degeneration and dysfunction [29]. Based on recent studies, insufficient amounts of BDNF promote neuroretinal apoptosis and degeneration. BDNF is a nerve growth factor and facilitates the repair and survival of ischemia-induced neural retina. It is also a key factor for maintaining interneurons connection in developing retina. BDNF can prohibit RGC apoptosis in rat models treated with ischemic or hypoxic conditions. BDNF binds specifically to TrkB, induces TrkB homodimerization, and establishes its downstream cascades. Two targets are proposed for TrkB including ERK/MAPK and PI3K/PKB. Both pathways are considered to be involved in BDNF-driven neuroprotection. TrkB is expressed in RGCs and has a high affinity to BDNF and NT-4 [8]. In hyperglycemia condition, BDNF causes overexpression and phosphorylation of TrkB and performs a neuroprotective role in the retina. ERK has been considered as the downstream candidate for BDNF stimulation in this condition.

In a study by Liu et al., it has been reported that BDNF neuroprotection in the retina is concentration-dependent. At supra-optimal doses in which BDNF is saturated, neuroprotection is not improved, perhaps due to a limited number of TrkB receptors. Additionally, the overdose of BDNF may result in retinal inflammation [8]. It has been demonstrated that BDNF can stimulate platelet reactivation and inflammation [34]. Studies in animal models have shown that the perpetual application of BDNF for intravitreal injection has a reducing effect on TrkB expression, which seems to be another reason for the dose-dependent activity of BDNF in the retina. BDNF can stimulate the expression of TrkB at both mRNA and protein levels. BDNF also increases the phosphorylation and activation of TrkB. In contrast to TrkB, BDNF does not affect the expression of ERK but induces its phosphorylation. According to this study, BDNF can protect neurons from hyperglycemia through TrkB and ERK/MAPK [8].

Different studies have been conducted to survey the effect of BDNF as a therapeutic option for RGC regeneration. These studies have been used in different animal models such as rats, mice, and rabbits. BDNF is a suitable target for mir365 mRNA. Age stress results in the overexpression of Mir365 mRNA which in turn decreases the level of BDNF mRNA. Lixia Lu et al. showed that in diabetic retina Mir365 mRNA increased in weeks 1-2 and subsequently BDNF was decreased in week 4 [53]. So it was suggested that Mir365 mRNA had a regulatory effect on BDNF. Following this observation, it was shown that anti-Mir365 mRNA could stop apoptosis of neural retina. Another approach for DR treatment is BDNF delivery to the eye. One of BDNF delivery strategies to the eye posterior segment is intravitreal injection. Recently, it was shown that BDNF intravitreal injection could prevent degeneration of dopaminergic amacrine cells [14]. Several studies have reported that the BDNF dose should be calculated precisely related to animal weight. Although a single injection could improve RGC survival a few weeks after injection, this effect did not last for a long time. This phenomenon may be due to the short half-life of BDNF protein [54]. Multiple injections could overcome this limitation but increased the risk of cataract, retinal ischemia, and endophthalmitis [55]. Another way to increase BDNF stability was the utilization of a more stable solution to dissolve BDNF such as 0.5% hyaluronic acid instead of phosphate buffer saline [22]. A combination of BDNF to a biological molecule before BDNF delivery could also prolong BDNF stability [23]. In recent years, some alternative ways of BDNF delivery to the eye were introduced such as viral mediated and cell-based mediated delivery. Adenovirus associated vectors among the others are considered to be the most appropriate for gene therapy in terms of long-lasting gene expression and safety [24, 25]. Muller cells and RGCs are the targets for gene transduction [56]. It seems that BDNF gene therapy is a way to omit unwanted side effects of intravitreal injection and in combination with intravitreal injection may yield an acceptable release of BDNF into the retina [57].

Another option for BDNF delivery is the intravitreal or subretinal injection of stem cells transfected with retroviral vectors [26–28, 58]. There are of course some complications with this protocol such as retinal folding, retinal detachment, and choroidal neovascularization [59]. The application of nanoparticles seems to be helpful for the delivery of BDNF and could compensate for BDNF delivery complications in the before-mentioned studies [54]. Some beneficial features of nanoparticle systems are controlled drug targeting and drug release [60]. Recently, it has been demonstrated that nanoporous silica nanoparticles could deliver BDNF to spiral ganglion neurons in a long-term period [61].

## 4. Conclusion

BDNF and VEGF are two important factors that should be under consideration for DR treatment. They have neuroprotective effects in the retina, can prevent apoptosis, and establish regeneration of RGCs in the retina. Recent evidence indicates that BDNF enhances VEGF-mediated angiogenesis; however, in DR patients, decreased level of BDNF is also associated with high VEGF secretion and retinal neovascularization. BDNF and VEGF appear to have a mutual relationship for tissue regeneration. Decreased level of serum BDNF stimulates neural degeneration and release of cytokines which ultimately leads to overproduction of VEGF. The new vessels produced by VEGF secretion provide the basis for the recruitment of BDNF to the retina and regeneration of neural retina. BDNF therapy in the early stage can delay neuroretinal degeneration and downregulate VEGF, but BDNF at high concentrations induces inflammation and exacerbation of angiogenesis. It seems that BDNF has a dose-dependent manner of neuroprotection and its dose should be precisely calculated regarding animal or model weight. BDNF seems to have its best neuroprotective effect in the early stages of DR and optimal concentration. Recent evidence suggests that, in the late stages of DR, severe damage to retinal cells leads to overproduction of VEGF and cytokines in retinal microenvironment which induces further inflammation resulting in the alteration of BDNF and VEGF receptors expression. We suggest that BDNF delivery to the eye is needed to be under precise control depending on the rate of retinal degeneration. Further research is certainly required to clarify the dose and period of BDNF prescription regarding the level of retinal degeneration in diabetic retinopathy.

## Figures and Tables

**Table 1:** Summary of reports focusing on the role of BDNF and VEGF in retinal neuro-vasculopathy and their clinical and experimental application for treatment.

Reference	Specimen understudy	Result/outcome
[2]	Serum samples of AMD patients and normal people	Higher BDNF levels in AMD group compared with normal controls.
[8]	Retinal neurons cell culture from postnatal Wistar rats	Protective role of BDNF in retinal neurons from hyperglycemia through TrkB/ERK/MAPK pathway.
[9]	Diabetic rats and postmortem eye specimens	Degeneration of retinal neural cells early in diabetic rats.
[10]	Vitreous samples from diabetic patients with DR	Decreased amount of inter-photoreceptor retinoid-binding protein in early human diabetic retinopathy and its association with retinal neurodegeneration.
[11]	Osteoblasts cell culture from Sprague-Dawley rats	BDNF promotes expression and secretion of VEGF from osteoblasts by TrkB/ERK1/2 signaling pathway.
[12]	Cultured RPE cells on the DAergic cells	RPE cells as sources of GDNF and BDNF secretion.
[13]	Serum and vitreous samples of DR patients and normal people	Decrease of BDNF in the serum and aqueous humor of diabetic patients before the emergence of clinical signs of retinopathy.
[14]	Streptozotocin-induced diabetic rat retinas	Decreased level of BDNF in early retinal neuropathy of diabetes. Intraocular administration of BDNF could protect dopaminergic amacrine cells from neurodegeneration.
[15]	Serum and vitreous samples of DR patients and normal people	Role of BDNF in controlling the pro- and anti-inflammatory cytokines. Association between low level of BDNF and development of diabetic retinopathy.
[16]	Adeno-associated virus (AAV) gene therapy (AAV2 TrkB-2A-mBDNF) in the inner retina of mouse and rat models.	Long-term increase of ERK and AKT signaling pathway in RGCs.
[17]	Molecular study of VEGF polymorphism	C (-634) G polymorphism in the 5′UTR of the VEGF gene is a novel genetic risk factor for diabetic retinopathy.
[18]	Laser-induced retinal ischemia in the monkey model	Upregulation of VEGF mRNA in ischemic retina.
[19]	Different cell cultures	Induction of VEGF and angiogenesis in hypoxia condition
[20]	Serum samples of DR patients receiving 30 mg Zn gluconate or maltose dextrin per day	Strong positive relationship between VEGF, BDNF, NGF, and their inverse association with metabolic factors was observed.
[21]	Serum and vitreous samples of DR patients and normal people	Increased amount of intravitreous VEGF and HIF-1a in diabetic patients with PDR.
[22]	Adult rat hippocampus-derived neural stem cells transplanted into the developing rat retina	Hyaluronic acid based BDNF could induce differentiation of grafted neural stem cells in rat retina.
[23]	Adult rats with elevated intraocular pressure	Combination of BDNF and LINGO-1-Fc prevented RGC death.
[24, 25]	Adenovirus-mediated gene transfer	Long-lasting and safe gene expression.
[26]	Retrovirus-engineered Schwann cell injected into the subretinal space of dystrophic RCS rats	Engineered Schwann cells sustain retinal structure and function in the dystrophic RCS rat.
[27]	DAPI-labeled BMSCs transplanted into the subretinal space of light-damaged Sprague-Dawley rats	BMSC subretinal transplantation could inhibit photoreceptor apoptosis and slow down retinal damage in light-damaged eyes.
[28]	rMSCs transduced with a retroviral vector expressing BDNF	Subretinal injection of rMSCs promoted rMSCs migration and incorporation into the rat retina and retinal BDNF mRNA and protein expression were increased at 4 weeks after transplantation.

## Abbreviation

BDNF: Brain-derived neurotrophic factor
VEGF: Vascular endothelial growth factor
DR: Diabetic retinopathy
AMD: Age-related macular degeneration
UTR: Untranslated region
rMSCs: rat bone marrow mesenchymal stem cells
BMSCs: Bone marrow stem cells
RGC: Retinal ganglion cell
RCS rats: Royal College of Surgeons (defective in their RPEs)
RPE: Retinal pigmented epithelium.
